# DTI-ALPS is not independently associated with overall survival beyond established prognostic factors in glioblastoma

**DOI:** 10.1007/s11060-026-05744-8

**Published:** 2026-08-06

**Authors:** Leon Schmidt, Sascha D. Santaniello, Harald Krenzlin, Andrea Kronfeld, Bianca Kollmann, Oliver Tüscher, Katrin Frauenknecht, Max Jägersberg, Sebastian Altmann, Marc A. Brockmann, Ahmed E. Othman

**Affiliations:** 1 Department of Neuroradiology, University Medical Center Mainz, Langenbeckstrasse 1, 55131 Mainz, Germany; 2 Department of Neurosurgery, University Medical Center Mainz, Mainz, Germany; 3 Institute of Neuropathology, University Medical Center Mainz, Mainz, Germany; 4 Department of Neurosurgery, University Hospital Schleswig-Holstein (UKSH) - Lübeck, Lübeck, Germany; 5 Leibniz Institute for Resilience Research (LIR) gGmbH, Mainz, Germany; 6 Department of Psychiatry, Psychotherapy and Psychosomatic Medicine, University Medicine of the Martin-Luther University Halle-Wittenberg, Halle, Germany

**Keywords:** Glioblastoma, Glymphatic system, DTI-ALPS, Extent of resection, Survival

## Abstract

**Purpose:**

Impaired glymphatic function has been associated with shorter survival in glioblastoma. However, the relationship between glymphatic dysfunction and survival has not yet been evaluated in the context of established prognostic factors, including extent of resection, adjuvant therapy, MGMT promoter methylation status, and clinical performance status.

**Methods:**

Single-center retrospective study including patients undergoing surgery for unifocal glioblastoma IDHwt CNS WHO grade 4 (GBM), with preoperative DTI studies in comparison to healthy controls (HC). Glymphatic impairment was assessed using diffusion tensor imaging analysis along the perivascular space (DTI-ALPS), which is an accepted surrogate marker for glymphatic function. This study follows the STROBE guidelines for cohort studies.

**Results:**

We included 47 patients with GBM and 50 HC. The hemispherically averaged ALPS index was lower in GBM patients (1.15 vs. 1.27, *p* = 0.01). There were no differences between contralateral ALPS and HC (1.22 vs. 1.27, *p* = 0.29). Ipsilateral ALPS was lower compared to HC (1.05 vs. 1.27, *p* < 0.001) and to the contralateral hemisphere (1.1 vs. 1.22, *p* = 0.01). Univariable Cox regression showed no association between overall survival and ALPS parameters (ipsilateral HR 2.25 95% CI 0.49–10.31 *p* = 0.3, contralateral HR 2.96 95% CI 0.57–15.32 *p* = 0.2). In multivariate analysis significant associations were found for female and extent of resection but not for MGMT status, age, ipsi- or contralateral ALPS indices.

**Conclusion:**

In patients with GBM, glymphatic function might be impaired as expressed by decreased ALPS index in the ipsilateral hemisphere. Ipsi- or contralateral ALPS index reduction did not predict overall survival independent of known predictors. Because DTI-ALPS has inherent limitations, further investigation of glymphatic dysfunction using different methods in glioblastoma is warranted.

**Supplementary Information:**

The online version contains supplementary material available at https://doi.org/10.1007/s11060-026-05744-8.

## Key points

DTI-ALPS method indicates impaired glymphatic function in the presence of glioblastoma in comparison to healthy individuals.

In our cohort, glymphatic impairment showed no association with reduced overall survival.

**Supplementary Information:**

The online version contains supplementary material available at https://doi.org/10.1007/s11060-026-05744-8.

## Importance of the study

The glymphatic system describes organized neurofluid exchange and perivascular drainage in the central nervous system and is proposed to play a role in immunosurveillance and tumor microenvironment. Prior studies suggest that impaired glymphatic function, assessed via the DTI-ALPS method, is associated with reduced survival in glioblastoma, apparently independent of tumor size, age and sex. However, the clinically most relevant prognostic factors MGMT status and extent of resection have not yet been assessed in this context. Our study is the first to include these in a multivariable survival analysis together with ALPS-derived metrics. Contrary to previous findings glymphatic impairment did not independently predict overall survival in our cohort. These findings highlight the necessity to include clinically important outcome predictors when assessing potential novel biomarkers in glioblastoma.

**Supplementary Information:**

The online version contains supplementary material available at https://doi.org/10.1007/s11060-026-05744-8.

## Introduction

Glioblastoma IDHwt (CNS WHO Grade 4) remains among the most challenging neoplasms, with prognosis remaining poor despite aggressive multimodal therapy. Established predictors of survival include molecular markers such as MGMT promoter methylation, IDH mutation status, extent of resection (EOR), and clinical performance status [1–3]. Beyond these factors, systemic alterations in the tumor microenvironment are of increasing scientific interest, yet in vivo assessment of contributing components remains limited [4, 5].

The glymphatic system describes organized neurofluid exchange through convective perivascular CSF influx, mediated by Aquaporin-4 channels on astrocytic end feet, with subsequent drainage towards meningeal lymphatic vessels and cervical lymph nodes. Beyond waste clearance and interstitial fluid homeostasis, the glymphatic system has been proposed to play a role in neuroinflammation and immunosurveillance, rendering it a potentially relevant contributor to the tumor microenvironment in glioblastoma [6, 7].

In vivo assessment of the glymphatic system is mainly performed via MRI-based techniques, including intrathecal or intravenous contrast agent application and diffusion imaging [8, 9]. Utilizing diffusion tensor imaging, the analysis along the perivascular space (DTI-ALPS) method quantifies diffusivity along perivascular spaces by calculating tensor direction orthogonally to known fiber tracts in the centrum semiovale, yielding the ALPS index as a surrogate for glymphatic function [10].

Previous studies have demonstrated impaired glymphatic function in glioblastoma via DTI-ALPS, with reduced indices correlating with shorter survival. However, the relationship between glymphatic dysfunction and the most clinically relevant survival predictors (EOR and MGMT methylation status) has not been examined. Furthermore, the validity of the ALPS index as a surrogate for glymphatic function in the presence of a localized infiltrative remains methodologically challenging, as tumor infiltration, edema and white matter disruption may influence diffusion-based measurements [8, 11–13]. Changes in the ALPS index are thus possible based on different biological changes, in the hemisphere ipsilateral to the tumor ALPS changes can either be attributed to tumor infiltration and disrupted brain microstructure or reduced diffusivity indicating glymphatic impairment. With less likely tumor infiltration in the contralateral hemisphere an association between glymphatic impairment and ALPS index is more likely in the contralateral hemisphere.

The aim of this study was to assess glymphatic function via DTI-ALPS in conjunction with established survival predictors including EOR and MGMT promoter methylation status in patients undergoing surgery for glioblastoma, with the goal of evaluating its potential as an independent prognostic biomarker.

## Methods

### Patients/participants

For the present study, we included patients who underwent resection for histologically confirmed glioblastoma IDHwt (CNS WHO Grade 4) at our institution between 1 January 2016 and 31 December 2025. The present study follows the STROBE guidelines for cohort studies. Patients were required to be over 18 years of age, to have histologic diagnosis of glioblastoma IDHwt (CNS WHO Grade 4), and to have preoperative DTI sequences available. Patients were excluded in case of severe image artifacts prohibiting analysis or if no anatomically suitable area for ALPS analysis was identified. Specifically in case of ROIs overlapping with contrast-enhancing tumor, patients were excluded. Patients who died during the initial hospital stay due to perioperative complications and non-tumor related causes were excluded from the survival analysis. For ALPS comparison, image data of healthy subjects participating in the AgeGain study with comparable imaging parameters were used [14].

### Clinical data

Demographic information, surgical- and treatment data was collected retrospectively from routine medical records, including electronic medical records. Data acquisition was concluded on 01 May 2026, patients alive at this time point were administratively censored at the date of the last follow-up.

### MRI acquisition and preprocessing

For anatomical images, standard T1-weighted MPRAGE sequences were performed. In patients with glioblastoma additional axial Flair and contrast-enhanced T1 MPRAGE sequences were acquired. Diffusion-weighted images (DTI) were acquired with 60–64 directions, b-values of 0 and 1000 s/mm² and phase encoding in A-P direction, for both glioblastoma patients and healthy controls. DTI sequences were routinely acquired when required for preoperative surgical planning.

### Radiographic measurements

Lesion volumes were calculated from routine preoperative MRI T1 contrast-enhanced and T2 flair sequences using SECTRA IDS7 (Version 27.1.13.8679, Sectra AB, Linköping, Sweden). Extent of resection was classified according to current RANO criteria based on the early postoperative MRI [15].

### ALPS index estimation

**Fig. 1:**
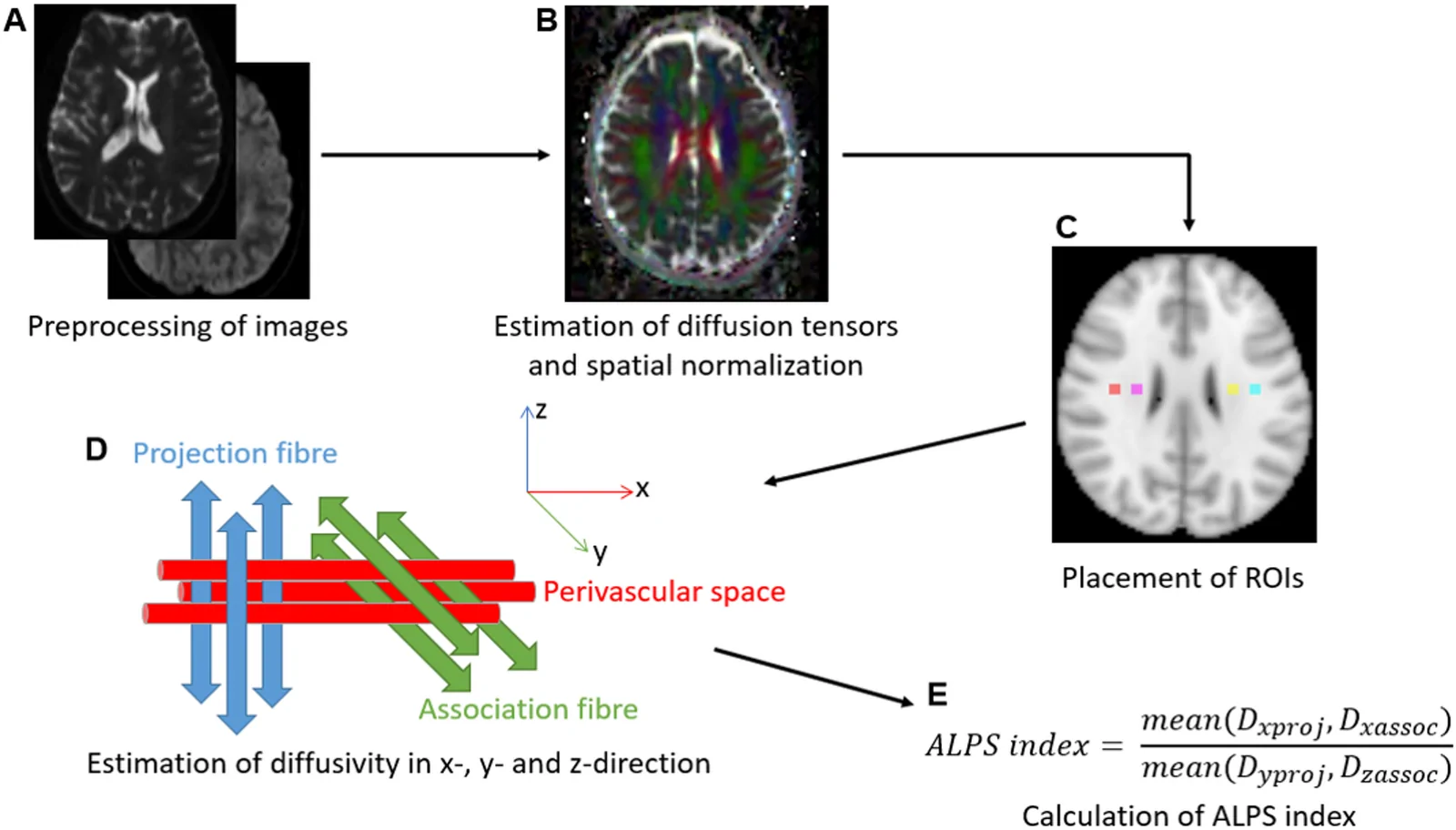
Graphical representation of the methodology used in this work. **A** Pre-processing of image data. **B** Spatial normalization to MNI-template and estimation of normalized DTI. **C** Placement of ROIs: The middle layer of the four ROIs used to determine the ALPS index values is shown. The ROIs are colored as follows: red: left superior longitudinal fasciculus (SLF), purple: left superior corona radiata (SCR), yellow: right SCR, light blue: right SLF. The SCR corresponds to projection fibers, the SLF to association fibers. **D** Estimation of diffusivity values in the selected ROIs. **E** Calculation of local GS index values

To evaluate GS characteristics, we calculated the ALPS index following the workflow implemented in our previous work, including preprocessing, motion correction and image registration for both groups [16]. Using the MNI152 template and the JHU-ICBM-Labels atlas, regions of interest (ROIs) corresponding to the superior longitudinal fascicle (SLF) and the superior corona radiata (SCR) were identified in both hemispheres. Within each region, a rectangular ROI consisting of four voxels was defined, yielding four ROIs. Thus ROIs were placed on a normalized template and automatically transferred to normalized patient imaging. Resulting ROI placement was reviewed to ensure that placement was acceptable in respect to SLF and SCR, no overlap with contrast enhancing tumor or different anatomical structures was present. ROI review was performed blinded to all clinical data and prior to ALPS calculation. For each ROI, the diffusion tensor and diffusivities along the x-, y-, and z-axes were calculated. The ALPS index was then derived according to the formula shown in Fig. 1E. Specifically, the mean diffusivity in the x-direction across both ROIs was divided by the mean diffusivity in the y-direction within the SCR ROI and in the z-direction within the SLF ROI, reflecting the orientation of projection fibers (SCR) and association fibers (SLF). The resulting values were used as a proxy for GS integrity. Hemispheric ALPS indices were computed separately for each brain side using the corresponding SLF and SCR ROIs, and the hemispherically averaged ALPS index was obtained by averaging values of both hemispheres. Additionally differences between ipsi- and contralateral ALPS index values as well as the lateralization index were calculated. ALPS values were not available to clinicians at any point of this study.

### Statistics

Absolute and relative frequencies were calculated for all categorical variables. Values were presented as median and interquartile range. Correlations were assessed using Spearman’s correlation coefficient. Group differences between independent samples were assessed using the Wilcoxon rank-sum test, while paired comparisons were performed using the Wilcoxon signed-rank test, or the paired t-test was utilized, as appropriate. For group comparisons of categorical variables, χ2 tests or Fisher´s exact test were used. Survival was analyzed using Kaplan-Meier analysis with log-rank tests and Cox proportional hazards regression. An optimal ALPS cut-off was determined using the maximally selected rank statistic. The proportional hazards assumption was tested using Schoenfeld residuals, linearity of continuous variables was tested using linear and spline-based Cox models using likelihood ratio tests. In the Cox models, ALPS indices and contrast-enhancing tumor volume were modeled as continuous variables, EOR was dichotomized according to the current RANO categories (classes 1–2 vs. classes 3–4). Statistical analysis was performed using R 4.5.2, R: A language and environment for statistical computing R Core Team (2026), Vienna, Austria (https://www.r-project.org).

### Ethics

According to the local laws of Rhineland Palatinate, Germany no formal approval and informed consent was necessary for this retrospective analysis. The patient data was anonymized before analysis.

## Results

### Demographics and baseline information

**Fig. 2:**
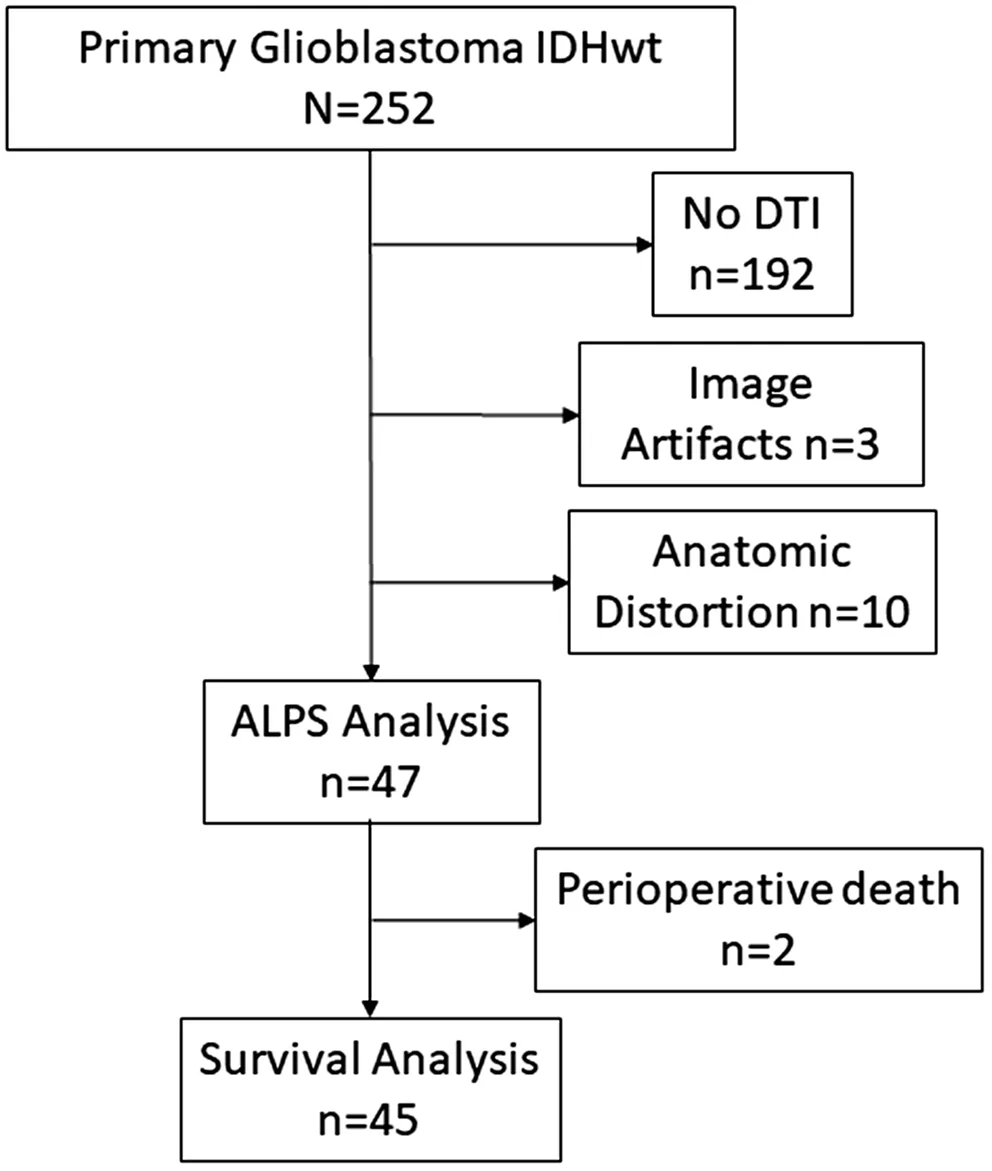
Flow diagram of patient selection and cohort assembly

From January 1, 2016, to December 31, 2025, sixty patients with unifocal glioblastoma IDHwt (CNS WHO Grade 4) underwent diffusion-tensor imaging and subsequent surgical resection. Imaging requirements for ALPS analysis, such as artefact-free images or possibility of ROI definition in WM without tumor-related displacement, were met in 47 cases. Exclusions are displayed Fig. 2. Of these 24 (51.1%) were female and 23 (48.9%) were male. Median age was 66 years (IQR 16.5 years). From the included healthy individuals 23 (46%) were female and 27 (54%) were male. Median age was 68.5 years (IQR 7 years). There were no differences in age or sex between groups (*p* = 0.18, *p* = 0.75). MGMT methylation was present in 23 tumors (48.9%). Midline shift was present in 15 (31.9%) cases, median contrast-enhancing tumor volume was 19.8 mL (IQR 28.8 mL), and median edema volume was 66 mL (IQR 96.8 mL).

All patients underwent surgical resection and early postoperative MRI. The extent of resection was classified according to current RANO criteria, with 7 (14.9%) achieving class 4, 6 (12.8%) class 3, 33 (70.2) class 2 and 1 (2.1%) class 1 resection. Subsequently 7 (14%) patients received no adjuvant treatment, 4 (9%) patients were treated according to the Nordic trial, 6 (13%) patients were treated analog to the Perry Regimen, 27 (58%) received Stupp Regimen and 3 (6%) patients received treatment analog to the CeTeG Regimen. Baseline and Treatment variables are further displayed in Table 1. No missing data was present in the included cohort.

**Table 1:** Baseline and treatment variables

	Glioblastoma	Healthy	
Number	47	50	
Female	24 (51.1%)	23 (46%)	*p* = 0.75
Age [years]	66(16.5)	68.5 (7)	*p* = 0.18
ALPS			
Hemispherical Average	1.15 (0.24)	1.27 (0.36)	*p* = 0.01
Ipsilateral	1.05 (0.24)		*p* = 0.29
Contralateral	1.22 (0.38)		*p* < 0.001
Tumor volume [ml]	19.8 (31.9)		
Edema volume [ml]	66 (96.8)		
Midline shift	15 (31.9%)		
MGMT methylation	23 (48.9%)		
Extent of Resection			
Class 4	7 (14.9%)		
Class 3	6 (12.8%)		
Class 2	33 (70.2%)		
Class 1	1 (2.1%)		
Adjuvant therapy			
None	7 (14%)		
Nordic	4 (9%)		
Perry	6 (13%)		
Stupp	27 (58%)		
CeTeG	3 (6%)		
OS [months]	15 (11–26)		

Values are presented as absolute numbers (%), median (IQR). Continuous variables were compared using two-sided Wilcoxon rank-sum tests, dichotomous variables were compared using Fisher’s exact test. Ipsilateral and contralateral ALPS indices within patients were compared using a two-sided paired Wilcoxon signed-rank test For ALPS index comparisons ipsi- and contralateral values were compared to the hemispherically averaged ALPS index of healthy individuals. OS: Overall survival

### ALPS index

**Fig. 3:**
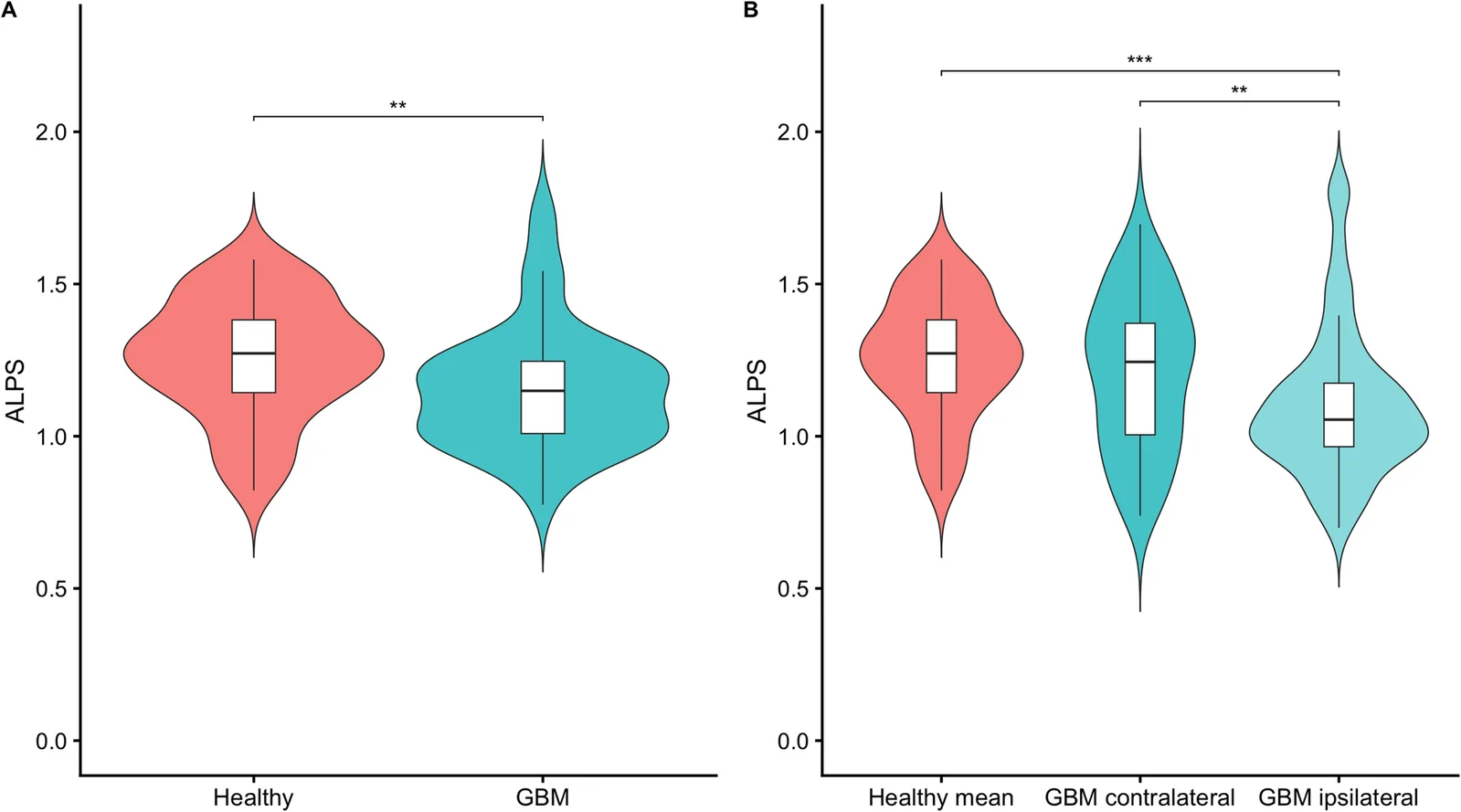
Distribution of ALPS indices between healthy individuals and patients with glioblastoma. **A** shows the difference in hemispherically averaged ALPS indices. **B** displays ipsi- and contralateral ALPS indices in comparison to the hemispherically averaged ALPS index of healthy controls. Group differences were assessed with the Wilcoxon rank-sum tests and paired Wilcoxon signed-rank tests for ipsilateral versus contralateral comparisons. GBM: glioblastoma** *p* < 0.01 *** *p* < 0.001

In patients with glioblastoma median ipsilateral ALPS was 1.05 (IQR 0.96–1.2), median contralateral ALPS was 1.22 (IQR 0.99–1.37), the median ALPS in healthy controls was 1.27 (IQR 1.03–1.39). The median hemispherically averaged ALPS Index was significantly lower in patients with glioblastoma than in healthy controls (1.15 vs. 1.27, t = 2.5, *p* = 0.01). There were no significant differences between ALPS index values in the contralateral hemisphere and healthy controls (t = 1.05, *p* = 0.29). Comparing ipsilateral ALPS and healthy controls revealed significantly lower values (W = 1697, *p* < 0.001). Additionally ipsilateral values were significantly lower than in the contralateral hemisphere (V = 339, *p* = 0.01). Distributions are displayed in Fig. 3. ALPS indices as well as hemispherical average, difference and lateralization index showed no significant correlation with age (ρ = 0.06 *p* = 0.67; ρ = 0.07 *p* = 0.71, ρ=−0.13 *p* = 0.38, ρ=-0.27 *p* = 0.07, ρ = 0.25 *p* = 0.09). Contrast enhancing tumor volume, edema volume and ECOG at admission or discharge did not correlate with ALPS metrics. No significant associations were observed between ALPS metrics and MGMT promoter methylation status, EOR, or chemotherapy regimen. MGMT methylation status was not associated with ALPS metrics. There were no differences of ipsi- or contralateral ALPS according to MGMT methylation status (*p* = 0.63, *p* = 0.06) between both groups.

### Survival

For the survival analysis two cases of perioperative deaths were excluded as these events were deemed not tumor related leaving 45 patients for survival analysis.

Median follow-up was 27 months (95% CI 16 months – not reached), during this period, 29 events were observed. Ten patients were censored at the last documented follow-up, while 6 were administratively censored at the end of data acquisition. Median overall survival was 15 months (95% CI 11–26 months). When comparing deceased patients to censored cases the only significant difference was median CE tumor volume (30.40 vs. 13.85 *p* = 0.025).

In Kaplan-Meier analysis only extent of resection was significantly associated with overall survival (*p* < 0.001). MGMT methylation failed to achieve a significance (*p* = 0.11).

**Fig. 4:**
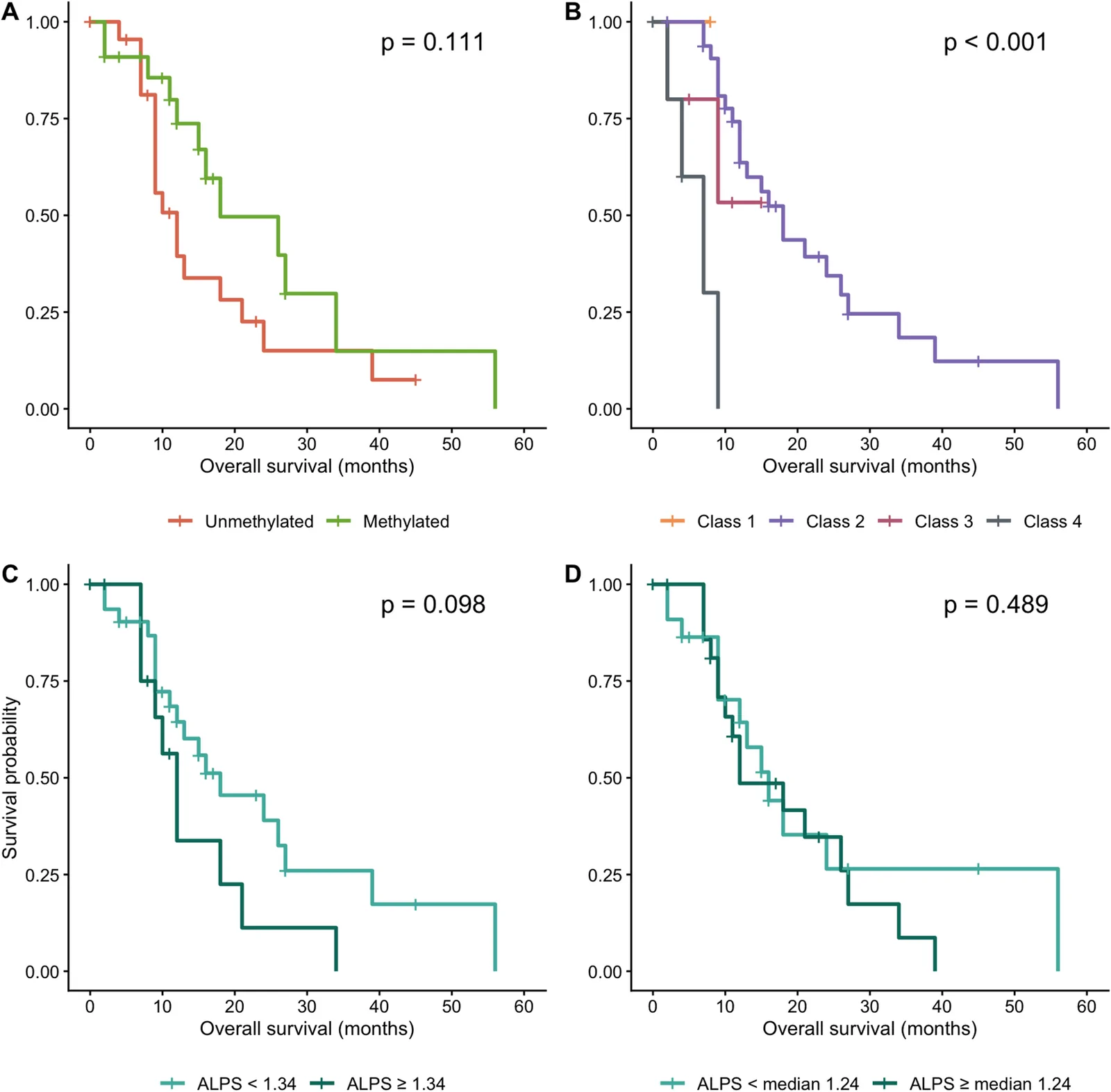
Kaplan-Meier curves for overall survival. **A** shows differences by contralateral ALPS dichotomized by the median **B** shows differences by contralateral ALPS dichotomized by the optimized internal cut-off of 1.34 **C** displays differences by MGMT methylation status and **D** displays differences by extent of resection by RANO classes. Survival distributions were compared using two-sided log-rank tests

For comparison with current literature we dichotomized ALPS values as above or below the median classifying 23 as above and 22 as below the median. Additionally we calculated an internal cut-off for the contralateral ALPS index to predict OS. The maximally selected rank statistic identified an internal cut-off of 1.336, resulting in 30 patients below and 15 patients at or above the cut-off. Kaplan-Meier curves are presented in Fig. 4. In univariable Cox regression neither the ipsilateral or contralateral ALPS index, nor the difference between both or the lateralization index significantly predicted overall survival (HR 2.25 95% CI 0.49–10.31 *p* = 0.3, HR 2.96 95% CI 0.57–15.32 *p* = 0.2, HR 1.18 95% CI 0.31–4.52 *p* = 0.8, HR 0.74 95% CI 0.04–14.27 *p* = 0.84). There were no significant associations with survival for both the median split (HR 1.3 95% CI 0.61–2.76 *p* = 0.5) and the calculated cut-off (HR 1.75 95% CI 0.81–3.79 *p* = 0.15).

**Fig. 5:**
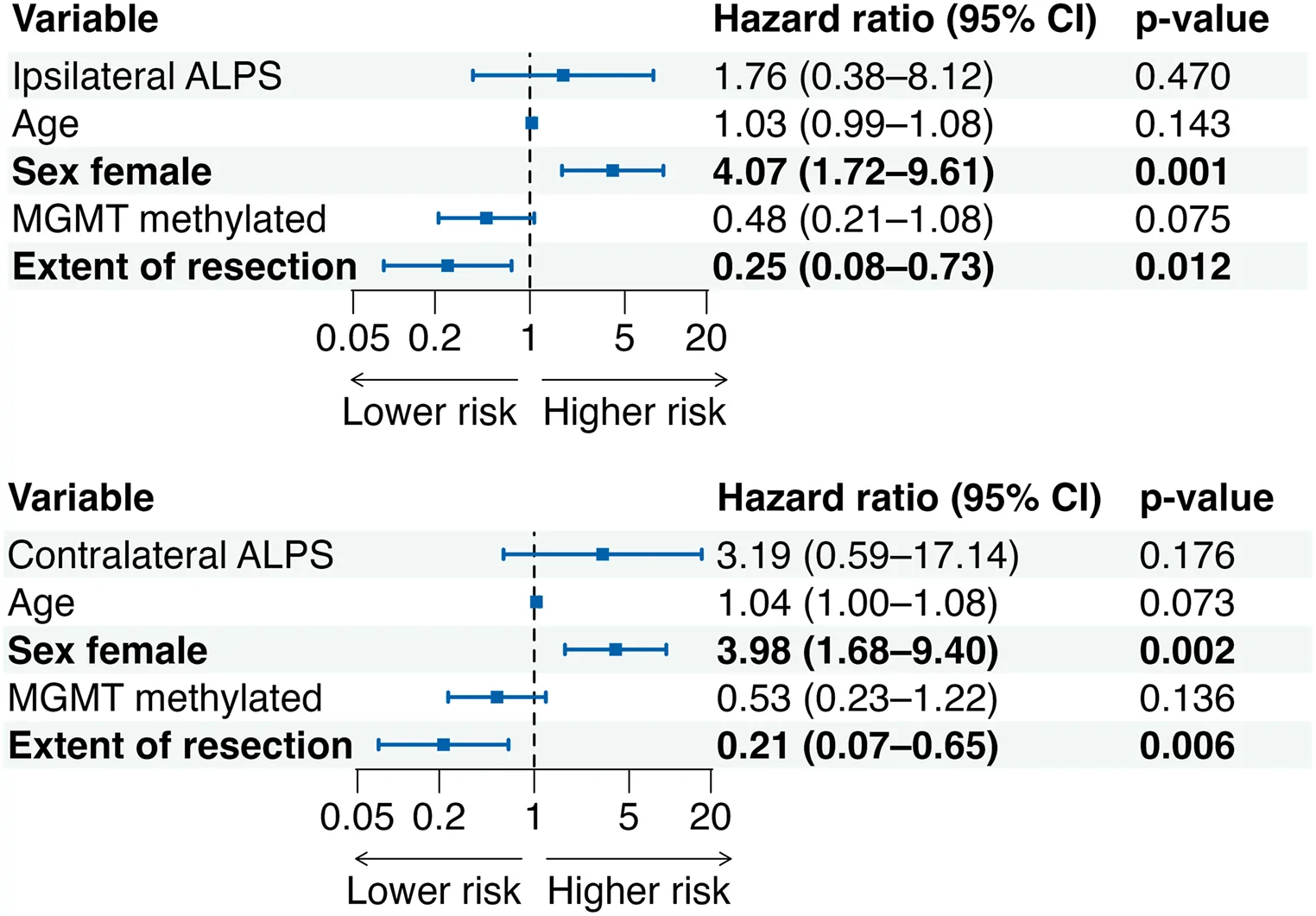
Forest plot of multivariable Cox regression analyses for overall survival. **A** Hazard ratios (HR) with 95% confidence intervals are shown for ipsilateral ALPS, age, female sex, extent of resection, and MGMT promoter methylation and **B** for contralateral ALPS, age, female sex, extent of resection, and MGMT promoter methylation. HR < 1 indicates a protective effect; HR > 1 indicates increased risk of mortality

Multivariable Cox regression models were used to assess independent association between ALPS indices and survival. Individual models additionally included age, sex, MGMT status and extent of resection. Ipsilateral ALPS was not independently associated with overall survival (HR 1.76 95% CI 0.38–8.12 *p* = 0.47), neither was contralateral ALPS (HR 3.19 95% CI 0.59–17.14 *p* = 0.18). In the model including the ipsilateral ALPS index significant predictors were female sex (HR 4.07 95% CI 1.72–9.61 *p* < 0.001) and extent of resection (HR 0.25 95% CI 0.08–0.73 *p* = 0.01). MGMT status was associated with longer OS but failed to reach significance (HR 0.48 95% CI 0.21–1.08), no association was observed for age (HR 1.03 95% CI 0.99–1.08 *p* = 0.14). When including the contralateral ALPS index in the model significant associations with survival were observed for female sex (HR 3.98 95% CI 1.68–9.4 *p* = 0.002) and extent of resection HR (0.21 95% CI 0.07–0.65 *p* = 0.006). Forest plots are displayed in Fig. 5. In order to prevent overfitting preoperative ECOG and adjuvant therapy were tested in additional sensitivity analyses. In therapy-adjusted analyses, both ALPS measures showed nominal associations with overall survival (ipsilateral HR 6.77 95% CI 1.07–42.77 *p* = 0.04; contralateral HR 9.67 95% CI 1.10–85.10 *p* = 0.04), but estimates were imprecise, and the associations were not retained after additional adjustment for ECOG performance status (Table S5). An analysis including the two perioperative events revealed no significant difference concerning these results. All primary models were statistically significant overall, with no evidence of violation of the proportional hazards assumption or non-linearity of continuous predictors. Global proportional-hazards tests were not estimable for the highly parameterized therapy-adjusted sensitivity models.

## Discussion

This study investigated the relationship between glymphatic dysfunction assessed using the established surrogate ALPS index and overall survival in relation to established survival predictors. Patients with glioblastoma in our cohort showed a significantly decreased ipsilateral ALPS index in comparison to healthy controls, while contralateral ALPS did not differ significantly from controls. This hemispheric asymmetry suggests that glymphatic impairment in glioblastoma might be predominantly driven by local tumor-related factors rather than a systemic effect. Our observation is consistent with a growing body of literature demonstrating impaired glymphatic function in glioma patients via DTI-ALPS [11, 12, 17–19]. These imaging findings are further supported by tracer studies in GBM rat models demonstrating reduced perivascular CSF flow and increased tracer retention [20].

In summary, our data further supports the hypothesis that glymphatic function might be impaired in the presence of glioblastoma and highlights the need to further understand the interactions between glioblastomas and the components comprising the glymphatic system. Nonetheless, the ALPS index currently relies on converging evidence for interpretation as indicating glymphatic dysfunction. Validation with tracer based studies in humans, assessing both methods, are currently lacking.

We did not observe an association between the ALPS index and overall survival. Overall survival was independently associated with sex and extent of resection, whereas age and MGMT status showed expected trends but did not consistently reach statistical significance. Besides the influence of sex on survival all factors are widely accepted prognostic markers in the current literature [1–3]. Concerning the strong association between sex and survival in the contralateral Cox model we were not able to identify imbalanced known outcome predictors possibly explaining this result. Because of the limited sample size, this may reflect residual confounding or random variation and should be interpreted cautiously.

In the key observation our data thus diverges from observations by Hagiwara et al., Tian et al. and Zeng et al. indicating ALPS as a prognostic factor for survival in glioblastoma [11, 12, 21]. While these studies assessed factors like age, sex, tumor volume and WHO grade, predictors like MGMT status and extent of resection were not assessed in conjunction with the ALPS index. To our knowledge, our study is the first reporting multivariable analyses including these highly relevant factors and the first to report a lack of association between ALPS-derived metrics and survival in glioblastoma. These negative findings may either be explained by cohort-specific characteristics, or the considerably smaller sample size compared with prior studies reducing our ability to detect a potentially modest effect on survival. Alternatively the failure to detect an association between the ALPS index and survival beyond established predictors might be attributed to a potential lack of additional prognostic information relayed by its assessment. It has to be noted that due to exclusion of patients with overlap of ROIs and tumor area a potential bias might be included as the patients with the probably most severe glymphatic impairment were not used for statistical analysis. However, there was no alternative but to exclude these, since the damaged fiber structure in the area of the tumor made it impossible to obtain reliable ALPS index values. The present multivariable analyses should therefore be considered exploratory and hypothesis-generating. To further assess the relevance of the ALPS index as a prognostic marker in gliomas it should be prospectively investigated in conjunction with known survival predictors. In glioblastoma, glymphatic dysfunction is likely a consequence of the tumor rather than a causal factor in disease progression [22]. This may account for the lack of prognostic value of the ALPS index observed in our study. These aspects warrant further longitudinal investigation.

When assessing the current body of evidence it is reasonable to conclude that glioblastoma induces changes in neurofluid dynamics that may extend beyond the immediate tumor surroundings, which raises the further need to understand glymphatic dysfunction as a contributor to the pathophysiology of high-grade gliomas. Recent scientific efforts to assess glymphatic function, especially in neurooncology, have heavily focused on assessing the ALPS index, but this method bears certain weaknesses. As it assesses diffusion metrics strictly within the corona radiata, it is anatomically constrained and reliant on microstructural white matter integrity [8, 13]. This exposes a vulnerability to confounding by tumor volume, tumor infiltration, vascularity and edema rendering it difficult to differentiate true glymphatic impairment from structural tissue changes.

In order to further understand glymphatic changes in glioblastoma additional investigations are needed. In our opinion efforts should include assessment of the ALPS index in larger cohorts, with harmonization of acquisition and processing protocols, in conjunction with known outcome predictors as well as methodologies possibly allowing for spatial assessment of glymphatic function like assessment of contrast clearance kinetics after i.v. or i.th. contrast application [23–25]. Future studies should further investigate whether EGFR alterations, TERT promoter mutation status, and Ki-67 proliferation indices modify the association between DTI-ALPS and clinical outcomes.

### Limitations

The presented study has some inherent limitations stemming from its retrospective design, which introduces a potential for selection bias. Most notably, eloquently located lesions may be overrepresented in our cohort, as DTI sequences are frequently requested for preoperative planning in these patients. Patients with glioblastoma frequently receive glucocorticoids to reduce edema which might potentially influence ALPS estimates, as medication information was not consistently available we were unable to assess a potential effect. Given the relatively small cohort size of 47 patients, the multivariable survival analysis should be interpreted with caution and requires validation in a larger prospective series. Because of the small sample size there is the possibility of incurring a Type II error, which in this context would mean that ALPS index is associated with survival in glioblastoma but our study failed to detect such an association. While image acquisition parameters were comparable between glioblastoma patients and healthy controls, potential bias related to scanner-specific effects cannot be completely excluded. The inherent limitations of DTI-ALPS as an indirect surrogate of glymphatic function are discussed in detail above.

## Conclusion

Glymphatic function assessed via DTI-ALPS appears to be reduced in the presence of glioblastoma but showed no association with overall survival in our cohort. Instead, established survival predictors like extent of resection remained independent prognostic factors. Given the inherent methodological limitations of the DTI-ALPS method, further investigation is needed to understand the mechanistic interactions between glioblastoma and glymphatic dysfunction.

## Supplementary Information

Below is the link to the electronic supplementary material.


Supplementary Material 1


## Data Availability

Data will be provided by the authors upon reasonable request via email to the corresponding author.

## Abbreviations

DTI: Diffusion Tensor Imaging
ALPS: Analysis along the Perivascular Space
OS: Overall survival
GBM: Glioblatoma IDH wildtype (CNS WHO Grade 4)
