# Supplementary material for: DTI-ALPS is not independently associated with overall survival beyond established prognostic factors in glioblastoma
Source: J Neurooncol. 2026 Aug 6;179(1):33. doi: 10.1007/s11060-026-05744-8 (PMC13447402; doi:10.1007/s11060-026-05744-8)
Supplement: Supplementary file 1 [file 11060_2026_5744_MOESM1_ESM.docx]

# Supplementary Material

Primary analysis cohort: n = 45; deaths = 29. Full cohort: n = 47; deaths = 31.

# Table S1. Associations between ALPS metrics and clinical variables

| **ALPS metric** | **Clinical_variable** | **Test** | **n** | **Effect** | **p-value** | **FDR-adjusted p** |
| --- | --- | --- | --- | --- | --- | --- |
| Ipsilateral ALPS | Age | Spearman rank correlation | 47 | ρ = 0.06 | 0.674 | 0.842 |
|  | ECOG at admission | Spearman rank correlation | 47 | ρ = -0.11 | 0.452 | 0.842 |
|  | Edema volume | Spearman rank correlation | 47 | ρ = 0.17 | 0.258 | 0.842 |
|  | Tumor volume | Spearman rank correlation | 47 | ρ = 0.17 | 0.266 | 0.842 |
|  | Chemotherapy protocol | Kruskal–Wallis | 47 | ε² = 0.00 | 0.890 | 0.993 |
|  | Extent of resection class | Kruskal–Wallis | 47 | ε² = 0.00 | 0.369 | 0.842 |
|  | Sex | Wilcoxon rank-sum | 47 | r = 0.04 | 0.814 | 0.842 |
|  | MGMT promoter methylation | Wilcoxon rank-sum | 47 | r = -0.036 | 0.831 | 0.842 |
|  | Midline shift | Wilcoxon rank-sum | 47 | r = -0.054 | 0.749 | 0.842 |
| Contralateral ALPS | Age | Spearman rank correlation | 47 | ρ = 0.067 | 0.715 | 0.842 |
|  | ECOG at admission | Spearman rank correlation | 47 | ρ = -0.21 | 0.164 | 0.842 |
|  | Edema volume | Spearman rank correlation | 47 | ρ = -0.12 | 0.445 | 0.842 |
|  | Tumor volume | Spearman rank correlation | 47 | ρ = -0.05 | 0.743 | 0.880 |
|  | Chemotherapy protocol | Kruskal–Wallis | 47 | ε² = 0.00 | 0.451 | 0.842 |
|  | Extent of resection class | Kruskal–Wallis | 47 | ε² = 0.03 | 0.223 | 0.842 |
|  | Sex | Wilcoxon rank-sum | 47 | r = 0.083 | 0.407 | 0.842 |
|  | MGMT promoter methylation | Wilcoxon rank-sum | 47 | r = -0.33 | 0.053 | 0.821 |
|  | Midline shift | Wilcoxon rank-sum | 47 | r = -0.121 | 0.508 | 0.842 |
| Hemispherically averaged ALPS | Age | Spearman rank correlation | 47 | ρ = -0.13 | 0.381 | 0.842 |
|  | ECOG at admission | Spearman rank correlation | 47 | ρ = -0.24 | 0.107 | 0.821 |
|  | Edema volume | Spearman rank correlation | 47 | ρ = -0.01 | 0.938 | 0.981 |
|  | Tumor volume | Spearman rank correlation | 47 | ρ = 0.03 | 0.838 | 0.937 |
|  | Chemotherapy protocol | Kruskal–Wallis | 47 | ε² = 0.00 | 0.669 | 0.842 |
|  | Extent of resection class | Kruskal–Wallis | 47 | ε² = 0.02 | 0.290 | 0.842 |
|  | Sex | Wilcoxon rank-sum | 47 | r = -0.225 | 0.187 | 0.880 |
|  | MGMT promoter methylation | Wilcoxon rank-sum | 47 | r = -0.079 | 0.664 | 0.821 |
|  | Midline shift | Wilcoxon rank-sum | 47 | r = -0.079 | 0.664 | 0.842 |
| ALPS difference | Age | Spearman rank correlation | 47 | ρ = -0.27 | 0.072 | 0.855 |
|  | ECOG at admission | Spearman rank correlation | 47 | ρ = 0.00 | 0.993 | 0.993 |
|  | Edema volume | Spearman rank correlation | 47 | ρ = -0.18 | 0.249 | 0.842 |
|  | Tumor volume | Spearman rank correlation | 47 | ρ = -0.08 | 0.581 | 0.842 |
|  | Chemotherapy protocol | Kruskal–Wallis | 47 | ε² = 0.00 | 0.853 | 0.937 |
|  | Extent of resection class | Kruskal–Wallis | 47 | ε² = 0.00 | 0.504 | 0.842 |
|  | Sex | Wilcoxon rank-sum | 47 | r = 0.014 | 0.932 | 0.993 |
|  | MGMT promoter methylation | Wilcoxon rank-sum | 47 | r = -0.254 | 0.136 | 0.842 |
|  | Midline shift | Wilcoxon rank-sum | 47 | r = -0.046 | 0.801 | 0.842 |
| ALPS lateralization index | Age | Spearman rank correlation | 47 | ρ = 0.25 | 0.091 | 0.821 |
|  | ECOG at admission | Spearman rank correlation | 47 | ρ = -0.01 | 0.973 | 0.993 |
|  | Edema volume | Spearman rank correlation | 47 | ρ = 0.18 | 0.243 | 0.842 |
|  | Tumor volume | Spearman rank correlation | 47 | ρ = 0.10 | 0.501 | 0.842 |
|  | Chemotherapy protocol | Kruskal–Wallis | 47 | ε² = 0.00 | 0.801 | 0.925 |
|  | Extent of resection class | Kruskal–Wallis | 47 | ε² = 0.00 | 0.484 | 0.842 |
|  | Sex | Wilcoxon rank-sum | 47 | r = -0.011 | 0.949 | 0.993 |
|  | MGMT promoter methylation | Wilcoxon rank-sum | 47 | r = 0.283 | 0.096 | 0.821 |
|  | Midline shift | Wilcoxon rank-sum | 47 | r = 0.029 | 0.873 | 0.842 |

Continuous clinical variables were assessed using Spearman rank correlations; two-group comparisons used two-sided Wilcoxon rank-sum tests, and variables with more than two groups used Kruskal–Wallis tests. P values were adjusted across all tests using the Benjamini–Hochberg procedure. Rank-biserial r and epsilon-squared (ε²) are reported as effect sizes.

# Table S2. Univariable Cox regression for overall survival

| **Predictor** | **n** | **Events** | **Hazard ratio (95% CI)** | **p-value** |
| --- | --- | --- | --- | --- |
| Ipsilateral ALPS | 45 | 29 | 2.25 (0.49–10.31) | 0.298 |
| Contralateral ALPS | 45 | 29 | 2.96 (0.57–15.32) | 0.195 |
| Hemispherically averaged ALPS | 45 | 29 | 4.15 (0.62–27.92) | 0.143 |
| Interhemispheric ALPS difference | 45 | 29 | 1.18 (0.31–4.52) | 0.813 |
| ALPS lateralization index | 45 | 29 | 0.74 (0.04–14.27) | 0.840 |
| Contralateral ALPS ≥ median | 45 | 29 | 1.30 (0.61–2.76) | 0.497 |
| Age, per year | 45 | 29 | 1.03 (0.99–1.06) | 0.179 |
| Gender Female | 45 | 29 | 2.74 (1.22–6.13) | 0.014 |
| MGMT Methylated | 45 | 29 | 0.53 (0.25–1.14) | 0.105 |
| EOR (Class 1 & 2) | 45 | 29 | 0.25 (0.09–0.68) | 0.006 |
| Tumor | 45 | 29 | 1.01 (1.00–1.02) | 0.185 |
| Edema | 45 | 29 | 1.00 (1.00–1.01) | 0.393 |
| ECOG performance status at admission | 45 | 29 | 1.06 (0.73–1.54) | 0.751 |

Hazard ratios were estimated using Cox proportional hazards regression. For categorical predictors, the first factor level is the reference. No multiplicity adjustment was applied.

# Table S3. Multivariable Cox regression models for overall survival

| **Model** | **Predictor** | **Hazard ratio (95% CI)** | **p-value** |
| --- | --- | --- | --- |
| Model 1: ipsilateral ALPS | Ipsilateral ALPS | 1.76 (0.38–8.12) | 0.470 |
|  | Age, per year | 1.03 (0.99–1.08) | 0.143 |
|  | Gender Female | 4.07 (1.72–9.61) | 0.001 |
|  | MGMT Methylated | 0.48 (0.21–1.08) | 0.075 |
|  | EOR (Class 1 & 2) | 0.25 (0.08–0.73) | 0.012 |
| Model 2: contralateral ALPS | Contralateral ALPS | 3.19 (0.59–17.14) | 0.176 |
|  | Age, per year | 1.04 (1.00–1.08) | 0.073 |
|  | Gender Female | 3.98 (1.68–9.40) | 0.002 |
|  | MGMT Methylated | 0.53 (0.23–1.22) | 0.136 |
|  | EOR (Class 1 & 2) | 0.21 (0.07–0.65) | 0.006 |

The two prespecified models included either ipsilateral or contralateral ALPS and were adjusted for age, sex, MGMT promoter methylation, and extent of resection. HR, hazard ratio; CI, confidence interval.

# Table S4. Model performance and diagnostics

| **Model** | **n** | **Events** | **Parameters** | **Log-likelihood** | **AIC** | **C-index (95% CI)** | **Likelihood-ratio χ² (df)** | **LR p** | **Wald p** | **Score p** | **Global PH p** | **Maximum \|dfbeta\|** |
| --- | --- | --- | --- | --- | --- | --- | --- | --- | --- | --- | --- | --- |
| Model 1: ipsilateral ALPS | 45 | 29 | 5 | -71.66 | 153.33 | 0.778 (0.687–0.869) | 21.04 (5) | <0.001 | <0.001 | <0.001 | 0.198 | 0.361 |
| Model 2: contralateral ALPS | 45 | 29 | 5 | -70.98 | 151.95 | 0.789 (0.707–0.871) | 22.41 (5) | <0.001 | <0.001 | <0.001 | 0.169 | 0.323 |

The C-index confidence interval uses the model-based standard error. The proportional hazards assumption was evaluated using scaled Schoenfeld residuals. Maximum absolute dfbeta is reported as an influence diagnostic.

# Table S5. Sensitivity analyses additionally accounting for ECOG performance status or therapy

| **Model** | **Predictor** | **n** | **Events** | **Parameters** | **AIC** | **Hazard ratio (95% CI)** | **p-value** |
| --- | --- | --- | --- | --- | --- | --- | --- |
| Ipsilateral ALPS + ECOG | Ipsilateral  ALPS | 45 | 29 | 6 | 154.35 | 1.19 (0.22–6.47) | 0.843 |
| Contralateral ALPS + ECOG | Contralateral  ALPS | 45 | 29 | 6 | 153.21 | 2.58 (0.45–14.64) | 0.286 |
| Ipsilateral ALPS + therapy | Ipsilateral  ALPS | 45 | 29 | 9 | 141.95 | 6.77 (1.07–42.77) | 0.042 |
| Contralateral ALPS + therapy | Contralateral  ALPS | 45 | 29 | 9 | 141.57 | 9.67 (1.10–85.10) | 0.041 |
| Ipsilateral ALPS + ECOG + therapy | Ipsilateral  ALPS | 45 | 29 | 10 | 143.65 | 4.91 (0.57–41.95) | 0.146 |
| Contralateral ALPS + ECOG + therapy | Contralateral  ALPS | 45 | 29 | 10 | 142.93 | 7.28 (0.72–73.41) | 0.092 |

Models additionally included ECOG performance status at admission, chemotherapy protocol, or both. These models are exploratory because the added variables increase the number of estimated parameters relative to the number of events.

# Table S6. Model performance and diagnostics for sensitivity analyses additionally accounting for ECOG performance status or therapy

| **Model** | **n** | **Events** | **Parameters** | **Log-likelihood** | **AIC** | **C-index (95% CI)** | **Likelihood-ratio χ² (df)** | **LR p** | **Wald p** | **Score p** | **Global PH p** | **Maximum \|dfbeta\|** | **All coefficients estimable** |
| --- | --- | --- | --- | --- | --- | --- | --- | --- | --- | --- | --- | --- | --- |
| Ipsilateral ALPS + ECOG | 45 | 29 | 6 | -71.18 | 154.35 | 0.791 (0.703–0.879) | 22.02 (6) | 0.001 | 0.001 | <0.001 | 0.109 | 0.345 | Yes |
| Contralateral ALPS + ECOG | 45 | 29 | 6 | -70.61 | 153.21 | 0.793 (0.709–0.876) | 23.15 (6) | <0.001 | <0.001 | <0.001 | 0.219 | 0.267 | Yes |
| Ipsilateral ALPS + therapy | 45 | 29 | 9 | -61.97 | 141.95 | 0.813 (0.715–0.911) | 40.42 (9) | <0.001 | 0.027 | <0.001 | Not estimable | 63259.928 | Yes |
| Contralateral ALPS + therapy | 45 | 29 | 9 | -61.78 | 141.57 | 0.836 (0.740–0.931) | 40.80 (9) | <0.001 | 0.028 | <0.001 | Not estimable | 57453.608 | Yes |
| Ipsilateral ALPS + ECOG + therapy | 45 | 29 | 10 | -61.83 | 143.65 | 0.821 (0.728–0.914) | 40.71 (10) | <0.001 | 0.033 | <0.001 | Not estimable | 24390.868 | Yes |
| Contralateral ALPS + ECOG + therapy | 45 | 29 | 10 | -61.46 | 142.93 | 0.837 (0.741–0.934) | 41.44 (10) | <0.001 | 0.014 | <0.001 | Not estimable | 5117.147 | Yes |

Model performance is reported using log-likelihood, Akaike information criterion (AIC), Harrell's concordance index (C-index), and global likelihood-ratio, Wald, and score tests. The proportional hazards assumption was assessed using scaled Schoenfeld residuals. Maximum absolute dfbeta is reported as an influence diagnostic. Models with non-finite coefficients were considered not fully estimable.

# Table S7. Sensitivity analysis including perioperative deaths

| **Model** | **n** | **Events** | **Predictor** | **Hazard ratio (95% CI)** | **p-value** |
| --- | --- | --- | --- | --- | --- |
| Ipsilateral ALPS | 47 | 31 | Ipsilateral ALPS | 1.76 (0.41–7.54) | 0.444 |
|  | 47 | 31 | Age, per year | 1.03 (0.99–1.08) | 0.130 |
|  | 47 | 31 | Gender Female | 4.54 (1.95–10.55) | <0.001 |
|  | 47 | 31 | MGMT Methylated | 0.51 (0.23–1.13) | 0.097 |
|  | 47 | 31 | EOR (Class 1 & 2) | 0.20 (0.07–0.56) | 0.002 |
| Contralateral ALPS | 47 | 31 | Contralateral ALPS | 2.20 (0.47–10.28) | 0.318 |
|  | 47 | 31 | Age, per year | 1.04 (1.00–1.08) | 0.076 |
|  | 47 | 31 | Gender Female | 4.58 (1.98–10.61) | <0.001 |
|  | 47 | 31 | MGMT Methylated | 0.55 (0.25–1.23) | 0.147 |
|  | 47 | 31 | EOR (Class 1 & 2) | 0.18 (0.06–0.50) | 0.001 |

Models included all patients, including deaths occurring within 30 days after surgery, and used the same covariates as the primary models.

# Table S8. Proportional hazards assumption

| **Model** | **Predictor** | **Scaled Schoenfeld χ²** | **df** | **p-value** |
| --- | --- | --- | --- | --- |
| Model 1: ipsilateral ALPS | Ipsilateral ALPS | 0.69 | 1 | 0.408 |
|  | Age, per year | 3.27 | 1 | 0.071 |
|  | Gender | 0.13 | 1 | 0.719 |
|  | MGMT | 0.10 | 1 | 0.754 |
|  | EOR | 2.31 | 1 | 0.129 |
|  | Global test | 7.31 | 5 | 0.198 |
| Model 2: contralateral ALPS | Contralateral ALPS | 2.98 | 1 | 0.084 |
|  | Age, per year | 2.71 | 1 | 0.100 |
|  | Gender | 0.54 | 1 | 0.463 |
|  | MGMT | 0.10 | 1 | 0.753 |
|  | EOR | 2.36 | 1 | 0.124 |
|  | Global test | 7.78 | 5 | 0.169 |

The proportional hazards assumption was assessed using scaled Schoenfeld residuals with the Kaplan–Meier time transformation. A non-significant p value indicates no evidence against proportional hazards.

# Table S9. Linearity assessment using restricted cubic splines

| **ALPS_metric** | **n** | **Events** | **Knots (10th, 50th, 90th percentiles)** | **Linear-model AIC** | **Spline-model AIC** | **Non-linearity LR χ²** | **p for non-linearity** |
| --- | --- | --- | --- | --- | --- | --- | --- |
| Ipsilateral ALPS | 45 | 29 | 0.826, 1.034, 1.375 | 153.33 | 154.64 | 0.69 | 0.407 |
| Contralateral ALPS | 45 | 29 | 0.843, 1.244, 1.552 | 151.95 | 153.94 | 0.01 | 0.916 |

Restricted cubic spline models used three knots at the 10th, 50th, and 90th percentiles. The likelihood-ratio test compares the spline model with the corresponding linear model; its p value tests non-linearity.

# Table S10. Median split and maximally selected rank statistic analyses

| **Analysis** | **Cut_off** | **Group sizes (below / at or above)** | **Hazard ratio (95% CI)** | **Cox p-value** | **Log-rank / adjusted maxstat p** |
| --- | --- | --- | --- | --- | --- |
| Median split | 1.244 | 22 / 23 | 1.30 (0.61–2.76) | 0.497 | 0.489 |
| Maximally selected rank statistic | 1.336 | 30 / 15 | 1.75 (0.81–3.79) | 0.153 | 0.958 |

The median split used the median contralateral ALPS value in the primary cohort. The maximally selected rank statistic used log-rank scores, candidate group proportions of 20%–80%, and the Lau94 p-value approximation. The adjusted maxstat p value accounts for cut-point selection.

# Table S11. Comparison of censored and deceased patients

| **Characteristic** | **Censored** | **Deceased** | **Test** | **p-value** |
| --- | --- | --- | --- | --- |
| Age, years | 64.5 [59.0–70.0] | 69.0 [58.0–75.0] | Wilcoxon rank-sum | 0.704 |
| Ipsilateral ALPS | 1.010 [0.858–1.157] | 1.053 [0.970–1.174] | Wilcoxon rank-sum | 0.281 |
| Contralateral ALPS | 1.128 [0.988–1.378] | 1.269 [1.054–1.379] | Wilcoxon rank-sum | 0.530 |
| Mean bilateral ALPS | 1.112 [0.973–1.249] | 1.149 [1.016–1.244] | Wilcoxon rank-sum | 0.470 |
| Tumor volume, cm³ | 13.8 [1.7–28.8] | 30.4 [18.1–48.9] | Wilcoxon rank-sum | 0.025 |
| Edema volume, cm³ | 49.5 [18.4–101.5] | 80.3 [53.4–132.0] | Wilcoxon rank-sum | 0.180 |
| ECOG at admission | 1.5 [1.0–2.0] | 1.0 [1.0–2.0] | Wilcoxon rank-sum | 0.880 |
| Sex |  |  | Fisher exact | 0.353 |
| Male | 10 (62.5%) | 13 (44.8%) |  |  |
| Female | 6 (37.5%) | 16 (55.2%) |  |  |
| MGMT promoter methylation |  |  | Fisher exact | 0.221 |
| Unmethylated | 6 (37.5%) | 17 (58.6%) |  |  |
| Methylated | 10 (62.5%) | 12 (41.4%) |  |  |
| Extent of resection |  |  | Fisher exact | 0.483 |
| No | 5 (31.2%) | 6 (20.7%) |  |  |
| Yes | 11 (68.8%) | 23 (79.3%) |  |  |
| Midline involvement |  |  | Fisher exact | 0.514 |
| No | 12 (75.0%) | 18 (62.1%) |  |  |
| Yes | 4 (25.0%) | 11 (37.9%) |  |  |
| Chemotherapy protocol |  |  | Fisher exact (Monte Carlo) | 0.258 |
| 0 | 1 (6.2%) | 3 (10.3%) |  |  |
| 1 | 3 (18.8%) | 1 (3.4%) |  |  |
| 2 | 1 (6.2%) | 5 (17.2%) |  |  |
| 3 | 9 (56.2%) | 19 (65.5%) |  |  |
| 4 | 2 (12.5%) | 1 (3.4%) |  |  |

Continuous variables are median [interquartile range] and were compared using two-sided Wilcoxon rank-sum tests. Categorical variables are n (%) and were compared using Fisher’s exact tests; Monte Carlo simulation was used for tables larger than 2 × 2.
